# Preclinical models of arthritis for studying immunotherapy and immune tolerance

**DOI:** 10.1136/annrheumdis-2021-220043

**Published:** 2021-08-11

**Authors:** Gavin R Meehan, Ranjeny Thomas, Shaima Al Khabouri, Pascale Wehr, Catharien MU Hilkens, David C Wraith, Daniela Sieghart, Michael Bonelli, György Nagy, Paul Garside, David F Tough, Huw D Lewis, James M Brewer

**Affiliations:** 1 Institute of Infection, Immunity and Inflammation, University of Glasgow, Glasgow, UK; 2 University of Queensland Diamantina Institute, The University of Queensland, Woolloongabba, Queensland, Australia; 3 Division of Rheumatology, Department of Medicine, Karolinska Institutet, Karolinska University Hospital, Stockholm, Sweden; 4 Center for Molecular Medicine, Karolinska University Hospital Solna, Stockholm, Sweden; 5 Translational & Clinical Research Institute, Newcastle University, Newcastle upon Tyne, UK; 6 Institute of Immunology and Immunotherapy, College of Medical and Dental Sciences, University of Birmingham, Birmingham, UK; 7 Division of Rheumatology, Department of Internal Medicine III, Medical University of Vienna, Vienna, Austria; 8 Department of Rheumatology & Clinical Immunology, Semmelweis University, Budapest, Hungary; 9 Department of Genetics, Cell and Immunobiology, Semmelweis University, Budapest, Hungary; 10 GlaxoSmithKline Research and Development, Stevenage, Hertfordshire, UK

**Keywords:** therapeutics, arthritis, experimental, arthritis, rheumatoid

## Abstract

Increasingly earlier identification of individuals at high risk of rheumatoid arthritis (RA) (eg, with autoantibodies and mild symptoms) improves the feasibility of preventing or curing disease. The use of antigen-specific immunotherapies to reinstate immunological self-tolerance represent a highly attractive strategy due to their potential to induce disease resolution, in contrast to existing approaches that require long-term treatment of underlying symptoms.

Preclinical animal models have been used to understand disease mechanisms and to evaluate novel immunotherapeutic approaches. However, models are required to understand critical processes supporting disease development such as the breach of self-tolerance that triggers autoimmunity and the progression from asymptomatic autoimmunity to joint pain and bone loss. These models would also be useful in evaluating the response to treatment in the pre-RA period.

This review proposes that focusing on immune processes contributing to initial disease induction rather than end-stage pathological consequences is essential to allow development and evaluation of novel immunotherapies for early intervention. We will describe and critique existing models in arthritis and the broader field of autoimmunity that may fulfil these criteria. We will also identify key gaps in our ability to study these processes in animal models, to highlight where further research should be targeted.

## Introduction

**Figure 1:**
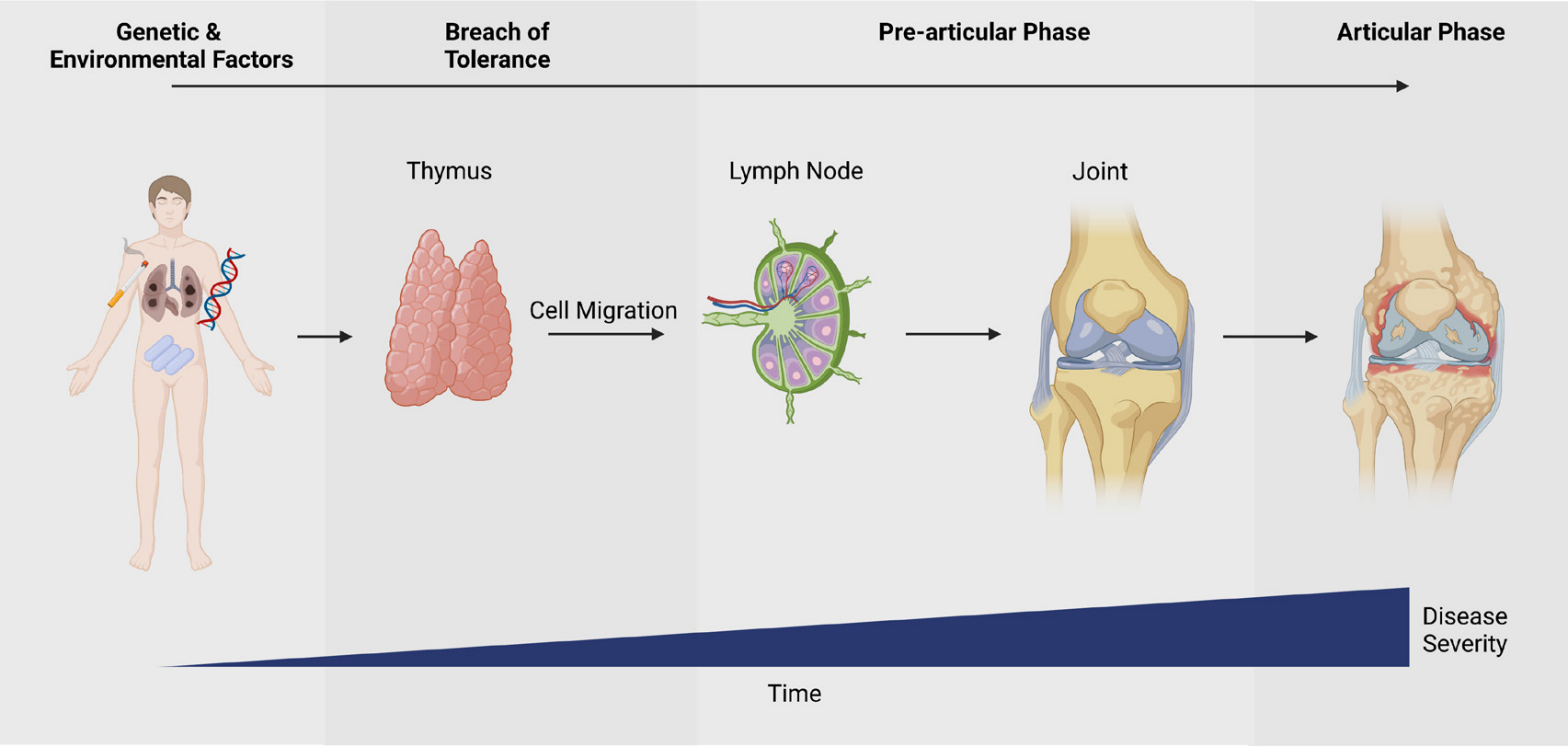
Disease progression of rheumatoid arthritis - created with BioRender.com.

Rheumatoid arthritis (RA) is a chronic inflammatory autoimmune disease that results in the destruction of the bone and cartilage of the joints. The disease is thought to be driven by genetic predisposition and environmental factors, leading to a loss of immunological self-tolerance, autoimmunity and arthritis (figure 1).

It is widely accepted that the combination of arthralgia and the presence of antibodies (indicating loss of tolerance) to citrullinated proteins (ACPAs) and or IgM rheumatoid factor (RF) is appropriate to identify individuals with high risk of developing RA.^1–4^ Approximately 30%–40% of subjects at risk will develop RA within 1 year. Several factors might indicate even higher risk: (1) high levels of ACPA (>three times of the upper level of normal) and/or RF (although RF is probably less important), (2) human leucocyte antigen (HLA) susceptibility alleles, such as shared epitope, (3) evidence of synovitis based on imaging (generally ultrasound and MRI), (4) smoking and (5) obesity. Based on all these factors individuals with up to 50%–60% risk to develop RA within 1 year might be identified.^5–7^ Disease progression to RA is associated with decreasing potential for remission.^8^ Treatment in the pre-RA phase might be associated with complete suppression of clinical signs and symptoms and the potential for the re-establishment of tolerance.^9^

Current treatments for RA consist of glucocorticoids, conventional and targeted synthetic and biological disease-modifying antirheumatic drugs (DMARDs). However, DMARDs decrease inflammation and ameliorate the radiological progression of the disease without altering the underlying pathology. The focus of recent autoimmune disease research has been to reinstate immunological self-tolerance. An ‘immunological reset’ with antigen-specific immunotherapy may ultimately allow for drug-free remission in RA, in essence curing the disease.

Arthritis research has employed a number of animal models which vary in their design and method of disease induction as well as the stage in the disease process they represent. The benefits of these models and their contributions to research have been discussed extensively in other reviews.^10–13^ Significantly not all models of RA are appropriate for the study of antigen-specific, tolerising immunotherapy.

Here, we focus on models that are suited to the study of initiating events in pre-RA (table 1) and are therefore well placed for identifying therapeutic targets for tolerance induction and for the resulting testing and development of new therapies. Importantly, we identify key questions about arthritis and how these models may contribute to our understanding of different immunological processes and antigen-specific immunotherapies.

**Table 1:** Preclinical arthritis models suitable for the development of tolerogenic therapies

Species	Induction of disease	Endogenous or exogenous antigen	Incidence rate (%)	Autoresponses involved	Is model synchronised?	Clinical course	Benefits for tolerance studies	Limitations for tolerance studies
Human	Spontaneous	Unknown	1% of population	Generation of autoantibodies (CII, ACPA, RF) and autoreactive T cells. Formation of immune complexes	–	Genetic susceptibility or environmental exposure, Breach of tolerance, prearticular phase, articular phase, chronic	–	–
Mouse (BALBc or C57BL/6J)	Immunisation with ovalbumin in mice that have ovalbumin specific T cells	Exogenous leading to endogenous response	80–100	Generation of autoantibodies (CII, ACPA, RF) and autoreactive T cells. Formation of immune complexes	Yes	Breach of tolerance, prearticular phase, acute but can be made chronic with further challenge	Congenic markers distinguish between OVA specific and endogenous T cells. True breach of tolerance. Autoreactivity only occurs with articular challenge	Inflammation is self resolving. Only polyarthritic with further challenge
Mouse (BALB/cAnNCrl)	Immunisation with Human cartilage proteoglycan aggrecan	Endogenous	100	Generation of autoantibodies (PG, ACPA, RF) and autoreactive T cells	Yes	Breach of tolerance, prearticular and articular phase, chronic	Tetramers are available to characterise antigens specific T cells.	Can only be used in BALB/cAnNCrl mice purchased from Charles River
Rat (various) or Mouse (BALBc, DBA/1, C57BL/10 or C57/BL6*)	Immunisation with bovine, murine or chicken collagen II	Exogenous/ Endogenous	70–100	Generation of autoantibodies (CII, ACPA, RF) and autoreactive T cells	No	Breach of tolerance, prearticular and articular phase, chronic	Tetramers are available to characterise antigens specific T cells. Useful for studying effect of tolerance on both B and T cell responses.	Difficult to perform in C57BL/6 J mice which limits genetic manipulation
Mouse (HLA-DR1 on C57BL6xB10 background	Immunisation of genetically predisposed mice with bovine or mouse collagen II	Exogenous/endogenous	70–100	Generation of autoantibodies (CII, ACPA, RF) and autoreactive T cells	No	Breach of tolerance, prearticular and articular phase, chronic	Tetramers are available to characterise antigens specific T cells. Uses HLA associated with human RA. TCR skewed to DR1 restricted collagen	Require mixed background mice as well as DR1 gene. Breeding can be challenging
Rat (DA) or mouse† (CBA/Igb, DBA/1 or BALBc)	Immunisation with the adjuvant pristane	Endogenous due to adjuvant exposure	50–80	Generation of autoantibodies (CarP, RNPs, RF) and autoreactive T cells	Yes	Prearticular and articular phase, chronic	Strongly T cell dependent	Mostly limited to rats so genetic manipulation difficult. Expensive.

*Must use chicken type II collagen.

†Delayed onset.

ACPA, antibodies (indicating loss of tolerance) to citrullinated proteins; CarP, carbamylated protein; CII, type II collagen; OVA, ovalbumin; PG, proteoglycan; RF, rheumatoid factor; RNP, ribonucleoprotein.

### Can animal models help us understand loss of tolerance leading to autoimmunity?

Breach of self-tolerance is a central and early step in the development of autoimmune disease. While the list of self and post-translationally modified antigens that are recognised by the host immune response is increasing,^14^ it remains unclear why responses are directed at these particular proteins, what are the circumstances that drive autoimmune responses to these antigens and why they evade mechanisms of central and peripheral tolerance in RA. Underlying factors associated with RA susceptibility include genetic predisposition as well as environmental factors including smoking, various infections, lung inflammation, periodontitis and changes in the microbiome, which contribute to the breach of self-tolerance at mucosal interfaces well before the development of joint inflammation.^15–18^ Animal models can play a critical role in identifying and isolating the environmental and genetic mechanisms that promote loss of tolerance. For example, in animal models with genetic predisposition to autoimmunity, such as the ZAP-70-mutant SKG mouse, in which altered T cell receptor (TCR) signalling leads to modified thymic selection, either an environmental stimulus or additional genetic lesion is required to initiate arthritis. Thus germ-free SKG mice fail to develop peripheral arthritis with a beta-glucan trigger, but they do develop spondylitis.^19 20^ SKG mice in a specific pathogen-free environment develop spontaneous arthritis when crossed to ZAP-70-deficient mice.^21^ Equally, models such as collagen-induced arthritis (CIA) or proteoglycan (PG)-induced arthritis (PgIA) require specific, susceptible, genetic strains of mice for induction of autoimmunity.^22^ It is worth nothing that while SKG mice have been instrumental for understanding underlying disease mechanisms, they have not been useful to date for studying antigen tolerisation strategies as few self-antigens have been elucidated.^23^

**Figure 2:**
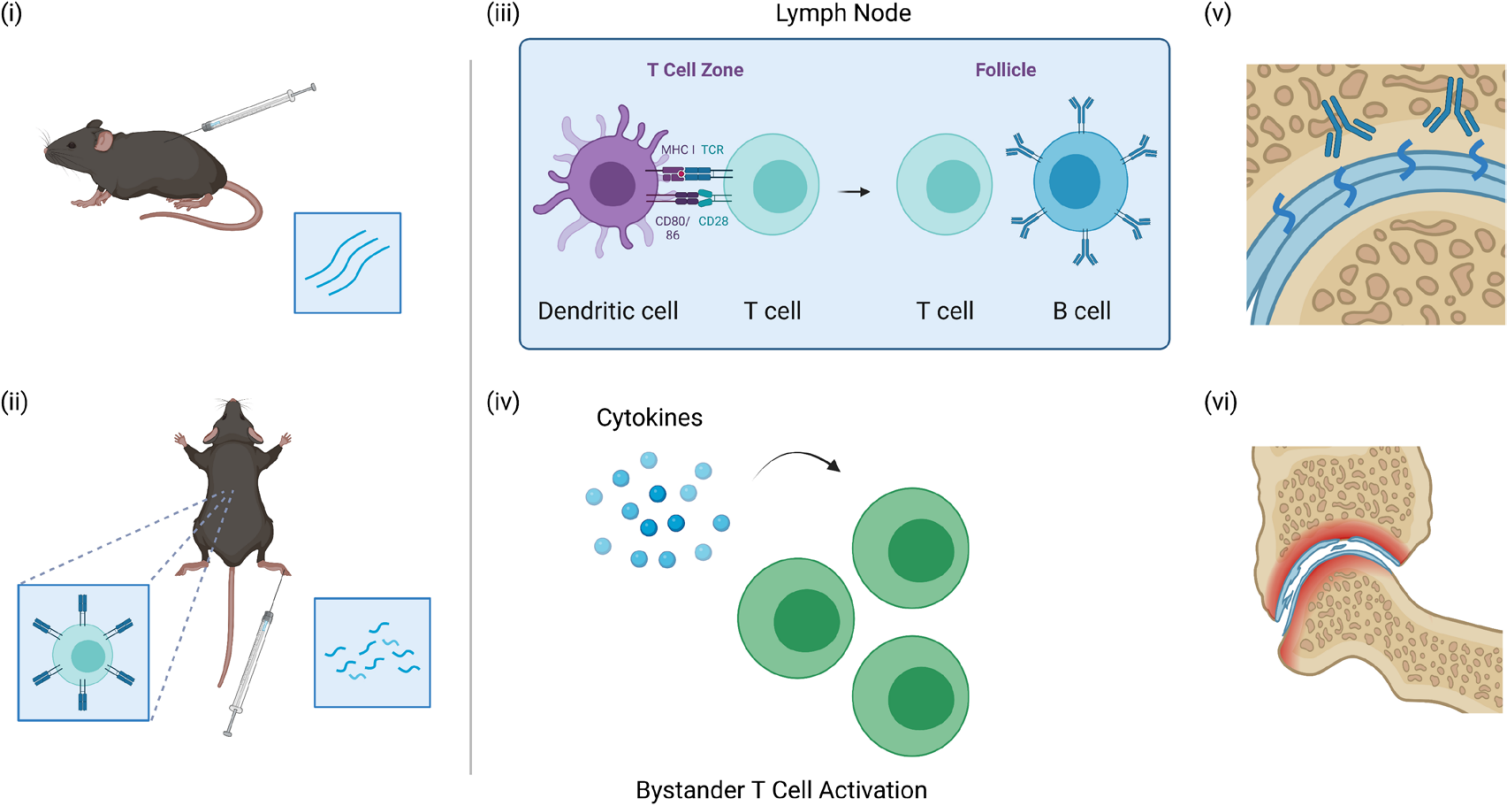
CIA and AIA models of arthritis. (i) CIA mice are injected with heterologous or autologous collagen in the presence of an adjuvant. (ii) in AIA models, mice are first immunised with an unrelated antigen in the presence of an adjuvant and then rechallenged with the same antigen in the joint. These models may employ the use of TCR transgenic T cells. (iii) The antigens in both models are initially presented by dendritic cells to CD4 T cells within the T cell zone of the lymph nodes. These CD4 T cells then interact with B cells within the follicle to produce antibodies. (iv) In the AIA models, the inflammation within the joint to the exogenous antigen triggers the activation of bystander T cells resulting in the targeting of joint antigens. (v) In both models, antigens within the joints become targeted by the immune response. (vi) This results in the destruction of cartilage and bones within the joints - created with BioRender.com. AIA, antigen-induced arthritis; CIA, collagen-induced arthritis; TCR, T cell receptor

In the CIA or PgIA models, a known antigen is administered to animals in the context of a powerful adjuvant, such as Freund’s complete adjuvant or dimethyldioctadecylammonium. This antigen is commonly a heterologous protein that closely resembles the endogenous protein of the animal, although models using autologous antigen have been demonstrated to also effectively induce arthritis in mice.^24 25^ In these models, the adjuvant creates an environment for immunogenicity of the antigen, inducing antibodies cross reacting with heterologous and endogenous antigen, leading to a loss of tolerance.^26^ While these mechanisms are well understood in CIA and PgIA models, they are unlikely to fully reflect how tolerance is breached in patients with RA, which is more complex, without a single initiating autoantigen with adjuvant, and involving the need for an ageing immune system to balance self-tolerance with immune control of micro-organisms. Other models of antigen-induced arthritis (AIA) using molecularly distinct antigens may help answer these questions (figure 2). In ovalbumin (OVA)-induced arthritis (OIA) or AIA, the eliciting antigen (OVA or methylated bovine serum albumin, respectively) is not an autoantigen; however, breach of self-tolerance occurs. This is instigated through the intra-articular injection of antigen into mice previously immunised with the same antigen and may employ the use of adoptively transferred antigen-specific T cells as in the OIA model. Following this challenge, there is a large influx of neutrophils and macrophages into the joint, resulting in the generation of B and T cells that recognise a range of unrelated autoantigens in addition to the initiating antigen (bystander activation).^27 28^ These latter two models allow closer analysis of the conditions that lead to autoimmunity as the bystander response to autoantigen can be considered ‘spontaneous’. Using this approach, the key role of cognate antigen (OVA) recognition in the joint and surrounding tissue was identified. Administration of either an inflammatory agent alone (lipopolysaccharides) or OVA subcutaneously is not sufficient to elicit autoimmunity.^29 30^ Further studies defined the role played by endogenous conventional dendritic cells (DC) in promoting breach of tolerance, as well as the regulatory role of plasmacytoid DCs.^31 32^ Future studies of these models will help define the range of autoantigens that are recognised in joint inflammation and, more importantly, when and why these particular host antigens are recognised and how they promote the process of epitope spreading. In this respect, it is important to note that immune recognition of post-translational modifications of endogenous proteins such as citrullination have been observed at low levels in some models,^29^ although there have been questions about the reproducibility of these results as well as the absence of appropriate controls.^33^ Whether ACPA are directly pathogenic in RA is still unclear. However, studies aimed at tolerising the T cell response to citrullinated antigens in both animals and humans may help define whether regulation of this response influences disease outcome.

While some models above contribute to our understanding of why breach of tolerance and autoimmunity develops, there remains considerable scope for improvement. Animal models offer the opportunity to perform reductionist approaches that allow dissection of the complex contributory genetic and environmental factors that lead to breach of tolerance. However, the mechanisms driving disease events in animal models do not necessarily replicate those occurring in human RA, for example, respiratory mucosal involvement, complex genetic background and contributory environmental factors, in addition to the long duration of disease. Furthermore, no spontaneous models faithfully reproduce human RA. Technologies such as animals expressing fluorescent reporters can be used to identify where and when key molecules are expressed, while cell-specific and tissue-specific gene knockouts can identify their mechanistic contributions to autoimmunity. These studies can be performed with the opportunity for the full temporal development of autoimmunity to be investigated, including assessment of where and when key therapeutic windows arise.

### Can animal models help us understand the progression from asymptomatic autoimmunity to joint infiltration and bone erosion?

The development of autoimmunity in RA and the transition into clinical disease remains a poorly understood process. Changes in innate immune reactivity and altered T cell and B cell regulation result in the development of autoantibodies targeting post-translationally modified proteins. These perturbations in immune cell activity indicate loss of tolerance and eventually culminate in the development of a synovial lesion that contains large numbers of infiltrating T cells, B cells, macrophages and fibroblasts.^34^

As this transitionary period generally occurs slowly over many years, different aspects of the immune response, particularly within the joints and lymph nodes, are difficult to study longitudinally in patients. Although animal models are unable to fully recapitulate human disease, their selective application has offered many insights into the development of autoimmunity and the complex interplay of immune cells in different tissues at various stages of disease. Importantly, as these models can be used in combination with technologies that would be otherwise impractical or unethical for use in patients, they allow for the study of discrete aspects of the disease that cannot be researched using other methods.

The ability to identify, manipulate and track specific cell populations is particularly useful in animal models, as has been shown in research examining the roles of autoreactive CD4 T cells in the development of early arthritis. The PgIA model has been used to demonstrate that TCR signalling strength dictates the fate of T cells, with those with weaker signals developing into T follicular helper cells (Tfh) which stimulate human PG-specific antibodies, cross-reactive with mouse PG.^35^ Since autoreactive T cells driving autoimmunity may have escaped central and peripheral tolerance mechanisms due to low TCR affinity, the fact that autoreactive T cells in RA mostly recognise modified self, which bind HLA with higher affinity, offers insights into the activation and persistence of Tfh and other effector cells driving autoimmune disease progression.

T cell migration studies, using multiphoton microscopy and lymph node sequestering drugs^36^ have also demonstrated that the majority of aggrecan-specific T cells are not involved in the pathogenesis of synovial inflammation directly, but rather exert their effects in the lymphoid organs where they provide B cell help for systemic autoantibody production.^37–39^ Similar work using a partially humanised CIA model in HLA-DR1 isotype (HLA-DR1) mice, in which chimeric human/mouse major histocompatibility complex (MHC) class II molecules comprise the peptide-binding domain from human DR and the CD4-binding domain derived from mouse I-E,^40 41^ has shown that T cells expressing an RA-relevant HLA-class II allele mount a response to the dominant epitope of collagen II. In this model, at the time of first clinical arthritis symptoms, specific effector CD4 T cells were undetectable in the synovial fluid and rare in the blood, but persisted in the lymph nodes.^42^

Taken together, data in PgIA and CIA models suggest that after the initial antigen-specific CD4 +T cell priming event in the lymphoid organs, disease development is dependent on B cells, which can present antigen and produce antibodies, and is perpetuated by CD4 Tfh cells which provide further B cell help for antibody-mediated joint destruction.^43 44^ Methods that disrupt Tfh and B cells within the lymph node may therefore offer a potential target for new immunotherapies.

Aside from T cells, animal models also implicate many other immune cells in arthritic disease development and regulation, including B cells,^45^ plasmacytoid DCs^31^ and synovial fibroblasts.^46^ Animal models offer major insights into immune cell dysfunction in arthritis. As new tolerogenic therapies are developed, antigen-driven animal models will be essential tools to understand how treatments impact immunological processes and will be key to understanding how these therapies function to restore immunological tolerance.

### How does the diversity of the TCR repertoire influence models?

TCR repertoire diversity is achieved on two levels: a genetic level involving selecting, editing and combining the various TCR genes, and on a cellular level involving thymic selection and outgrowth of certain clones in both acute and chronic immune responses. The strong association of autoimmune diseases, including RA, with certain HLA alleles is well documented.^47–49^ Thus, it is plausible that thymic selection and peripheral antigen encounter could influence the composition of the mature T cell repertoire in persons susceptible to RA and in patients with RA.^50 51^ Indeed, the outgrowth or enrichment of certain T cell clones has been demonstrated in RA, both in the naïve^52^ and antigen-experienced T cell compartments^53–56^ suggesting that both thymic selection and antigen-driven responses skew the TCR repertoire in patients with RA. Similarly in the CIA model in DBA/1 mice, the *IAq* allele is required for development of the disease due to high affinity binding of the collagen II dominant epitope to I-Aq after processing of collagen II protein, driving activation of autoreactive T cells.^57 58^

Moreover, TCR repertoire diversity in patients with RA differs depending on the tissues sampled. For instance, the repertoire was found to be more restricted in the synovial compartment compared with peripheral blood in patients with RA,^53 54 59 60^ indicating that tissue sites may influence the retention or accumulation of CD4 T cells possibly in an antigen-specific manner. TCR diversity has also been found to evolve with RA chronicity. In some cases, the TCR repertoire was more restricted in early RA and diversified with the progression of the disease,^54^ while in other cases the TCR repertoire was found to become more restricted with time.^61^ Additionally, changes in the TCR repertoire can also indicate patient response to therapeutics. For instance, patients treated with tumour necrosis factor inhibitors showed a reduction in clonal expansion in T cells expressing certain TCRβ variable region (TCRBV) genes,^62^ while responders and non-responders to methotrexate display differences in TCRBV gene expression profiles in the circulating CD4 T cell repertoire.^63^

The differences in TCR repertoire diversity reported at various stages of RA development and between different tissue sites highlights how assessment of TCR repertoire diversity has the potential of being an informative indicator of disease state and predictor of effective therapeutic regimens. However, patient to patient variability in clonal responses and the conflicting evidence of repertoire changes with disease progression accentuate our lack of understanding of how TCR repertoire diversity develops in RA and how it evolves with disease progression. Thus, animal models of arthritis can help elucidate development of the TCR repertoire as they provide a setting in which different disease stages can be observed more easily and allow for spatial and temporal assessment of TCR diversity.^62 63^ In addition, mouse models, such as CIA, with known dominant epitope, restricting I-A and HLA-DR molecules and responding T cells that can be identified with pMHC tetramers, offer a major advantage for T cell tolerance studies.

Models already exist that incorporate the influence of thymic selection on susceptibility to develop arthritis. For example, C57BL/6N.Q mice are more susceptible to CIA compared with C57BL/6 mice due to differences in MHC restriction^64 65^ and changes in T cell positive and negative selection in the SKG transgenic mice result in spontaneous development of arthritis.^23^ Studies examining aspects of TCR repertoire diversity have been conducted using the CIA model of arthritis and have also reported a skewed or restricted TCR repertoire and the prevalence of certain TCRβ chains were found to be strain dependent.^66–69^ The dominance of these chains were also relevant to the pathology as administration of depleting antibodies specific to the dominant Vβ chains were found to significantly reduce the incidence of CIA. One study using the HLA-DR1 mouse/CIA model found CD4 T cells of limited clonality in the joint with a highly selective subset of the TCR repertoire.^70^ These CD4 T cells bind to the dominant collagen II epitope and, although they comprise a minor population, they may play a major role in disease pathogenesis. A recent study investigated differences in the composition of the TCR repertoire in joints and their draining lymph nodes with the progression of OIA.^71^ The authors reported a disparity in TCR repertoire diversity between the draining lymph nodes and joints with the progression of inflammatory arthritis, with the lymph nodes displaying greater repertoire diversity than the joints at later stages of the disease. The results of the study highlight two main therapeutic implications; first, that tolerogenic therapies may be more effective at the very early stages of arthritis when the TCR repertoire is more restricted and, second, that TCR repertoire of joint-draining lymph nodes could possibly foreshadow TCR repertoire diversity of the joint, and thus be a marker of disease severity and guide effective therapeutic interventions. Significantly, animal models provide the opportunity to test these hypotheses, and rationalise the application of antigen-specific immunotherapy in disease.

### Are particular models more suitable for studying specific immunotherapeutic approaches?

There is a wide range of animal models available for arthritis research but not all models are well suited for studying tolerogenic immunotherapies. As these therapies can take many different forms it is essential that models are selected with consideration given to the method of tolerance induction. Optimising model selection will strengthen the data garnered from these studies and should improve the translation of this research into effective clinical treatments.

In the pathogenesis of RA, DCs act as key players in the development of autoimmunity as they, along with medullary thymic epithelial cells, present self-antigens to T cells in the thymus impacting negative selection, and in the periphery they are able to prime naive autoreactive T cells to initiate autoimmune models.^72^ However, DCs are also capable of inducing and maintaining peripheral tolerance by blocking T cell expansion, inducing T cell deletion or anergy. One promising cell-based approach is targeting autoreactive T lymphocytes by the production of tolerogenic DCs (TolDCs). The tolerogenic function of DCs can be promoted by the exposure to different anti-inflammatory cytokines or by in vitro treatment with an NF-kB inhibitor. TolDCs act by different mechanisms including the secretion of immunomodulatory mediators, reduction of MHC and costimulatory molecules or the expression of immune-modulatory/immune-inhibitory molecules.^73^ Preclinical data informing current clinical trials of TolDC immunotherapy in RA were derived from the ‘classical’ RA models, namely CIA^74–76^ and AIA models.^77^ Humanised mouse models of RA show several advantages in testing tolerogenic therapy by enabling direct translation to humans through introduction of human transgenes or by the selective transfer of human autoantigens or cells/tissue into immunodeficient mice.^78^ However, limitations include relatively poor expression of the human HLA transgene, and the need for induction of inflammatory arthritis with heterologous antigen, which limit interpretation of antigen presentation and efficacy of tolerising immunotherapies.^79^

The induction of regulatory T cells (Treg) by peptide-based therapies have been developed for the treatment of a number of autoimmune diseases including RA,^80^ multiple sclerosis (MS) and Graves’ disease.^81–83^ In this treatment, known tolerogenic peptides bind directly to MHC II on DCs.^84^ These DCs then interact with CD4 T cells to induce regulatory T cells that suppress T cell activation. As this therapy is based on peptide presentation, HLA-DR transgenic mice have supported the design of tolerogenic T cell epitopes and testing of tolerogenic strategies^85–87^; however, important lessons have been learnt. For example, introduction of a human HLA allele does not guarantee that an HLA-DR transgenic mouse will respond to an epitope known to be dominant in humans.^88^ This implies that mice have a ‘hole’ in their T cell repertoire for certain HLA-restricted T cell epitopes which can be overcome by creation of mice expressing both HLA-DR and TCR molecules from relevant patients.^85 89^ Furthermore, design work with individual peptide epitopes has shown that they must mimic naturally processed epitopes when bound to MHC II in order to induce tolerance through induction of IL-10 secreting regulatory T cells.^90 91^ This research confirms the importance of HLA-DR mice for the development and testing of peptide-based therapies in RA.

In addition to antigen-specific immunomodulatory therapy targeting DCs or T cells in situ, chimeric antigen receptor (CAR)-Treg cell therapy, in which Tregs are engineered to target specific proteins in a MHC independent manner,^92 93^ is being expanded to include autoimmunity in light of promising results from clinical trials, and product registration of CAR-T in oncology.^94^ In the context of RA and the HLA-DR1 model, it has been reasoned that engineering CAR-Tregs to specifically target an antigen in the joints of patients with RA may promote their migration to the site of abnormal inflammation, inducing a localised and protective immunosuppressive response. Accordingly, a CAR directed against citrullinated vimentin, a cytoskeletal protein, which is expressed in the synovial tissue of the majority of patients with RA, has been developed.^95^ This group is working to transduce this CAR into Tregs in order to assess functional activity in vitro and therapeutic potential in vivo of CAR-Treg transfer in the CIA model. Another approach in development is the generation of CAR CD8 CTL presenting antigenic peptide to specifically target and eliminate autoreactive CD4 T cells (Rosloniec, unpublished); these will also be tested in the HLA-DR1 humanised mouse model of CIA. While the CAR-Treg approach is advantageous in that it offers specific targeting and imparts no HLA restriction, its drawback is the requirement for a specific antigen for recognition, which is a design issue in RA due to the number of potential autoantigens involved in disease progression. Strategies invoking bystander tolerance or patient stratification based on putative autoantigen involvement and disease stage may facilitate therapeutic selection of CAR-T cell therapy to complement immunomodulatory approaches such as antigen-specific immunotherapies, as have been used in solid tumours in vivo in mice^96^

One of the oldest and most widely explored tolerogenic therapies is antigen feeding. In this therapy, small amounts of a specific antigen are administered orally to restore a state of homeostasis and tolerance to self-peptides in the adaptive immune system. This method has been used extensively with antigen-induced models, particularly CIA. Multiple experiments demonstrated that feeding collagen II prior to disease induction was protective against CIA in rats.^97 98^ Unfortunately, subsequent clinical trials with patients with RA showed conflicting results,^99–101^ with greater success observed with administration of lower antigen doses leading to the generation of active suppression via Tregs rather than anergy or clonal deletion.^102^ Due to inconsistencies between trials, this therapy was not pursued in RA. The disparity between animal models and clinical studies may lie in the lack of knowledge about the initiating autoantigen in RA, as collagen II is just one of many possible autoantigens involved in disease progression. Similarly, the timing of clinical trials of antigen feeding may be too late when autoimmunity has already progressed to disease. In addition, differences in rodent and human immune responses have to be considered.^103^ Despite these setbacks, antigen-induced CIA, OIA and AIA models are certainly useful to understand the mechanisms of how tolerance is induced from an immunological perspective. They may also offer insights into how antigen dosing and the timing of intervention affects disease outcomes.

DC targeting with antigen in the context of suppressing their activation is an emerging immunotherapy that is gaining popularity. DC targeting recapitulates models in which transgenic antigen targeted to ‘resting’ DCs promotes long-lasting peripheral tolerance through mechanisms of T cell deletion or regulation.^104^ Nanoparticles such as liposomes encapsulating disease-specific peptides along with immunomodulatory drugs, such as curcumin or calcitriol to suppress NF-kB activation required for DC activation, are taken up by DCs that interact with antigen-specific CD4 T cells to suppress disease progression.^105^ In the PgIA model, tolerising liposomes were found to significantly suppress disease severity.^106^ Peptide/calcitriol liposomes were found to exert their effects primarily through the deletion of high affinity antigen-specific autoreactive CD4 T cells and through anergy induction in the residual antigen-specific T cells. Delivery of the tolerising liposomes after the onset of disease also significantly reduced disease severity, even though arthritis is predominantly driven by autoantibody and complement-driven mechanisms in established disease.^107^ In contrast to pretreatment, the liposomes in this experiment were found to exert their effects through the expansion of FoxP3 +and IL-10-producing Tregs. Interestingly, this model suggests that the mechanisms of tolerance induction are dependent on the timing of liposome administration.

### Will current animal models identify where and when to intervene?

One of the major strengths of animal models of RA is that they allow for in-depth investigation of molecular and cellular processes at all different disease stages, that is, from initiation to chronic inflammation. They, therefore, also provide a powerful tool for studying immunotherapies, addressing important questions relating to the timing, route and frequency of administration and therapeutic effects. For example, using a rat allotransplantation model it was found that the timing and frequency of mesenchymal stem cell administration was crucial for graft survival, with multiple administrations having the best outcome in terms of the number of circulating Tregs.^108^ Similarly, administration of IL-4-transduced DC in CIA mice via the intravenous or intraperitoneal routes led to higher numbers of DC migration to the spleen and correlated with enhanced therapeutic effects as compared with the subcutaneous administration route.^109^

The disease stage is particularly important for immunomodulatory tolerance induction strategies, which use Tregs. The function, survival and stability of these cells is highly influenced by inflammation and tissue-specific factors which will vary depending on the stage and activity of the disease.^110^ Functional adaptation of FoxP3 +Tregs, also referred to as Treg plasticity, is an important process that occurs during protective immune responses. For example, exposure of Tregs to polarising cytokines directs expression of appropriate chemokine receptors that allow Tregs to home to and regulate the relevant site of inflammation. However, chronic exposure of Tregs to inflammatory mediators, as might occur, for example, in active RA, can backfire by destabilising FoxP3 expression and turn Tregs into pathogenic effector T cells. Indeed, it was shown that synovial fibroblast-derived IL-6 converted FoxP3 Tregs into Th17 cells with potent osteoclastogenic function in a CIA mouse model.^111^ This has important implications for Treg-based therapies, whether it is through adoptive transfer of Tregs, induction of FoxP3 +Tregs via adoptive transfer of tolerogenic DCs or in vivo expansion of existing Tregs with low dose IL-2, which shows promise in lupus as well as other autoimmune diseases.^112^ To avoid a detrimental conversion of Tregs, further investigation is required to optimise the timing of administration of tolerogenic immunotherapies, the potential for coadministration of anti-inflammatory drugs that could prevent Treg conversion (eg, anti-IL-6), and strategies and conditions that support or induce stable type 1 (Tr1) Treg from memory T cells.

Conversely, it is important to consider potentially adverse effects of existing RA medications on tolerance induction. For example, mouse models have shown that the calcineurin inhibitor ciclosporin A interferes with induction of allograft tolerance,^113^ and Cox-2 inhibitors (a subclass of non-steroidal anti-inflammatory drugs) inhibit oral tolerance to dietary antigens.^114^ The inhibitory effect of ciclosporin A is most likely caused by inhibition of Treg expansion and function.^115–117^ Testing the *in vivo* effects of relevant RA drugs on performance of tolerogenic therapeutics in preclinical animal models is important to determine the most suitable patient group for recruitment to clinical trials, and which DMARDs might help or hinder the tolerogenic response.

Another important question is where protolerogenic therapies should act. There is ample evidence that peripheral tolerance is chiefly induced in secondary lymphoid tissues—the same site as for priming of tissue-specific T cell clones. For example, immune tolerance to inhaled or oral antigens relied on CCR7-dependent migration of DCs to the relevant draining lymph nodes,^118 119^ and induction of allograft tolerance through treatment with IL-10-producing DCs also depended on CCR7-mediated homing of these DCs to the lymph node.^120^ It is not surprising that secondary lymphoid tissues play an important role in both immunogenic and tolerogenic immune responses, given that DCs (both mature and immature ‘tolerogenic’ DCs) as well as naïve T cells and Tregs home to these locations, providing the optimal architecture relevant for DC/T cell interactions. However, it is still uncertain whether this precludes the possibility that tolerance could be induced in different locations, for example, ectopic lymphoid structures at sites of inflammation (eg, in the rheumatoid joint) as with infiltrating Tregs that control tissue-destructive tumour-infiltrating lymphocytes in tumour sites.^121^ Understanding at which sites tolerance induction is most effective or even possible is critical to determine and to develop technologies for the most optimal routes of tolerogenic antigen (eg, TolDC) administration. Addressing these questions in humans is a major challenge. Although studies are underway to compare different routes of TolDC administration (intradermal vs intranodal) in the RESTORE study in patients with MS,^122^ and intradermal versus intra-articular versus intranodal in the AuToDeCRA2 study in patients with RA (Isaacs and Hilkens, unpublished), partially humanised animal models could aid in investigating these questions in more depth. For example, animal models provide an excellent tool for the longitudinal tracking and visualisation of interactions between different cell populations in vivo, including PET combined with vascular or lymphotracking dyes and CT or MRI, as well as multicolour fluorescence imaging. In some circumstances, these can be translated to clinical trials. Animal models can therefore be hugely beneficial in getting important clues on when and where to intervene, allowing for the improved, informed design of future clinical trials in patients with RA.

## Conclusion

**Figure 3:**
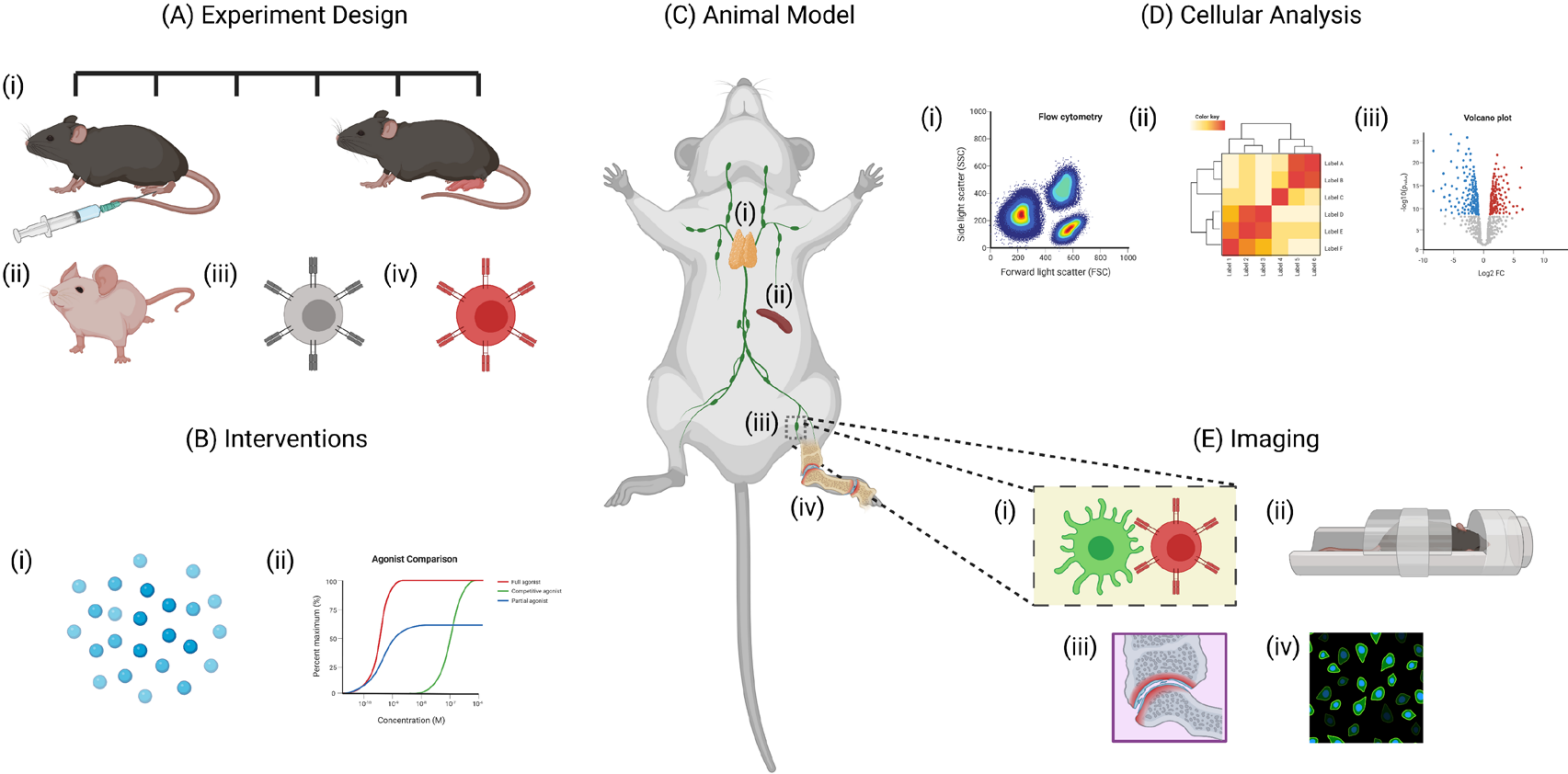
Benefits of using animal models for studying rheumatoid arthritis. Animal models allow researchers to study various aspects the disease that would otherwise be impractical to study in human patients. (A)(i)The experimental design of animal models allow researchers to monitor disease progression at various time points. Specific aspects of the disease can also be examined through the use of (ii) transgenic animals, (iii) TCR transgenic T cells and (iv) fluorescently labelled cells. (B) Interventions including (i) antigen-specific immunotherapies and (ii) drug treatments can also be studied in detail. (C) Tissues including the (i) thymus, (ii) spleen, (iii) lymph nodes and (iv) synovial tissue can be collected from animals at any time point. (D) This allows for detailed analysis of various cell populations using techniques such as (i) flow cytometry, (ii) RNA sequencing and (iii) cytokine assays. (E) Another major advantage of animal models is the use of live imaging techniques including (i) intravital imaging using multiphoton microscopy and (ii) whole tissue imaging using techniques such as MRI scanners. Similarly, tissues collected from culled animals can be imaged by (iii) histology or (iv) immunofluorescence - created with BioRender.com.

Although there have been many criticisms of animal models due to the poor translatability of data from preclinical models to clinical trials,^123^ currently these models remain essential to develop curative therapy in RA. Understandably not all aspects of human disease can be fully recapitulated in animal models including the long transition from breach of tolerance to autoimmunity as well as the extensive interplay of genetic and environmental factors that trigger the onset of disease. Despite these drawbacks, when proficiently applied in combination with different technologies, and selected to reflect appropriate points in disease progression, animal models are critical tools in mechanistic arthritis research and remain essential for the development of curative therapies (figure 3).

A key point is that of reverse translation. As new antigen-specific immunotherapies are developed, it is critical that data from clinical studies further inform model selection. This will allow for a targeted approach to research in animal models, where bioassays or technologies can be improved for future trials, and to identify the immunological mechanisms underlying human disease and therapeutic responses. Used in this way, animal models will facilitate the development and testing of new therapeutic agents to reinstate immunological self-tolerance.
